# Progress in Ophthalmology

**Published:** 1903-05-30

**Authors:** 


### Progress in Ophthalmology.

(*Concluded from page* 100.)

**Plastic Artificial Vitreous in Mules' Operation.**—Oatman,[3] of Brooklyn, in a paper read before the New York Academy of Medicine says: After the removal of an eye, the efforts of the ophthalmologist are put forward to mitigate, so far as possible, the subsequent effects of the operation. If an enucleation has been performed, the orbital contents will subsequently shrink so as to form but a poor support for the artificial eye. The severed ocular muscles having nothing on which to exercise their power, will atrophy. Little or no motion remains in the stump, while the immovable glass shell but emphasises the defect it was designed to conceal. The play of all the facial muscles is so infinitely associated with the movements of the eye that when the influence of the ocular muscles has been eliminated by enucleation, that side of the face will soon become flat and expressionless. Concerning Mules' operation, which consists of putting a hollow glass ball into the empty sclerotic, after removing the cornea, iris, and ordinary contents, and sewing the edges of the sclerotic together, he says when the operation is successful the result is ideal; but it was discovered that sooner or later this artificial vitreous was very apt to be extruded, the final result being similar to that obtained by an evisceration. Balls made from all sorts of material have been substituted for those of glass, but the results have not been materially modified, their final expulsion appearing to depend upon conditions other than their composition. When from any cause a fistulous opening has once formed over an artificial vitreous, it resists all attempts at repair until the foreign body has been expelled. Under these circumstances, he thinks that if a portion of the ball could be removed, and the tension on the scleral walls relieved, we might hope for closure of the wound over the remainder. Paraffin was selected as the most available material for carrying out this principle. In his first case the Mules' operation was performed as usual, except that instead of glass, a ball of paraffin was used to fill the vitreous cavity. The paraffin had a melting point of 120° F. The wound apparently closed satisfactorily, but it was soon discovered that a small fistula existed through which the paraffin was oozing. With a Daviel spoon warmed in hot water, a considerable qantity of the paraffin was easily extracted through the opening, the edges of which were then freshened and sutured. No success attended this procedure; the fistula remained patent, and the paraffin continued to escape. Two subsequent attempts to close the fistula were followed by failure, and it was decided that paraffin was unsuitable for this purpose. No change for the better occurred until the paraffin had been discharged down to the level of the fistula, which was about the centre of the ball. When this point had been reached and about half the paraffin had leaked away, the opening closed spontaneously, and has remained closed ever since, a period of about ten months. The remaining paraffin forms an excellent cushion for a shell and the stump has good motility. It was suggested that the paraffin would be absorbed gradually, but he says there is no apparent diminution in the size of the ball up till the time of writing. In preparing the ball the paraffin was first brought to the boiling point, and then cooled very quickly to prevent crystallisation or the formation of bubbles. A ball was then shaped roughly with a knife. It was placed on the point of a long needle and brought to the required size by rotating it on the flame of a spirit lamp, the action of the flame being controlled by dipping occasionally into a cold bichloride solution. He quotes two other cases, in one of which the paraffin escaped similarly to his own case, but in the other there was no escape, and after four months the result was quite good. In the first of these two cases the melting point of the paraffin used was 135° F., but even this did not prevent some escaping. Oatman recommends that silk sutures be used for the sclerotic, and that they be put in so close together that no aperture remains in the wound through which the paraffin may exude. A plastic material like paraffin will adapt itself to any inequalities on the inner surface of the glass eye, and ulceration from pressure is not apt to occur. Paraffin beads are easily prepared, and may be used in special cases in which glass beads of the required shape or size are not obtainable.

**Cosmetic Value of Paraffin Injections after Enucleation of the Eyeball.**—Maitland Ramsey[4] has a series of 22 cases to quote from, and it is interesting, coming after the former article on this subject. Of course, in this procedure, the eyeball is enucleated and the paraffin injected into the capsule of Tenon—a much more difficult procedure than merely inserting a paraffin ball into the empty sclera. He describes his operation as follows:—The conjunctiva is divided as close as possible to the corneal margin; each rectus muscle is caught up on a strabismus hook and sutured to the overlying conjunctiva with a strand of catgut; the tendons of the recti muscles are cut at their insertion into the sclerotic, and thereafter the operation for removal of the eyeball is completed in the ordinary manner. If adrenalin chloride solution be freely used the amount of bleeding is very slight, and the hæmorrhage is easily stopped by douching the socket with hot sterilised water. The capsule of Tenon is packed with gauze till a strong black silk purse-suture has been passed round the conjunctival margin, and then, the packing being removed, the melted paraffin is injected with a carefully sterilised glass syringe, the capsule being opened to its utmost capacity by holding the recti muscles on the stretch by means of the four cat-gut sutures and filled to overflowing. The purse suture is then tightened (care being taken to hold the patient's head steady and relax the tension on the recti muscles) and is securely fixed by a double knot, and the catgut sutures are tied, the superior rectus muscle being approximated to the inferior and the internal rectus to the external. The paraffin is thus induced to mould itself in the socket and to form a stump to which the divided muscles soon attach themselves. The excess is wiped away and after the conjunctival surface has been carefully bathed with boric solution, a compress and bandage are applied. This operation, he says, is followed by very little inflammatory reaction, and as a rule causes the patient hardly any more discomfort than simple enucleation does. The purse suture is kept in place for a fortnight, and when it is removed at the end of that time there will be found over the freely movable paraffin stump a clean non-discharging surface of conjunctiva. A week later an artificial eye can be adjusted, the ordinary shell proving as a rule quite satisfactory, though sometimes a better result may be obtained by using the form recommended by Snelien. To insure success, two points he thinks, require attention. Firstly, the operation must be carried out with every precaution against sepsis, and so it must not be attempted in cases where the eyeball is in a state of active suppuration. Secondly, the sutures must hold the conjunctiva in accurate position over the paraffin. It is on the purse suture that most reliance requires to be placed, and it is therefore very important to see when this is introduced that an equal grip is taken of the conjunctiva all round the free edge, and also, that too wide an interval is not left between the stitches, but the catgut strands to which the muscles are attached afford great additional security. In one of his recent cases in which the suture holding the internal rectus broke, a little opening made its appearance on the seventh day after the operation opposite the attachment of that muscle, and the aperture grew gradually larger and larger till the paraffin escaped. In his series of 22 cases there were four failures. Three times the paraffin came out through inefficiency of the stitching, but in two of these the conjunctiva was simply drawn together by the sutures holding the muscles, without any continuous suture. The purse suture was only introduced after the fourth case, and since then the only occasion in which the stitches have given way was the one referred to where the suture securing the internal rectus broke. On one occasion after the enucleation of a suppurating eyeball, pus apppeared in the socket and set up so much inflammatory reaction that the stitches were removed and the moulded paraffin allowed to escape. The paraffin employed is a sterilized preparation melting at 40° C., specially prepared by Rogers, Oxford Street, London. It seems simply to lie in the tissues and gives rise to no irritation, but whether it will become absorbed or not he is prepared to leave to time to show. Up to the present none of his cases have shown any sign of either shrinkage of the stump or impaired mobility. Both these methods seem to be excellent, but if the first can be accomplished one cannot help thinking the result would be much better as the muscles with their sclerotic attachment are not at all interfered with and would be more likely to contract and so perform their allotted task with greater facility.

[3] Med. Rec., March 7, 1903. [4] Lancet, Jan. 31, 1903.

